# Characterization of a genomic Island carrying the *tet*(X4) gene in porcine *Acinetobacter towneri* co-harboring plasmid-borne *bla*_NDM−1_ and *bla*_OXA−58_ genes

**DOI:** 10.3389/fvets.2022.1002149

**Published:** 2022-09-29

**Authors:** Aijuan Li, Runhao Yu, Wenbo Zhao, Stefan Schwarz, Chenglong Li, Hong Yao, Xiang-Dang Du

**Affiliations:** ^1^College of Veterinary Medicine, Henan Agricultural University, Zhengzhou, China; ^2^Department of Veterinary Medicine, Centre for Infection Medicine, Institute of Microbiology and Epizootics, Freie Universität Berlin, Berlin, Germany; ^3^Veterinary Centre of Resistance Research (TZR), Freie Universität Berlin, Berlin, Germany

**Keywords:** tigecycline, carbapenem, resistance, *tet*(X4), *bla*_NDM−1_, *bla*_OXA−58_, *Acinetobacter towneri*

## Abstract

Tigecycline and carbapenems are last-resort antimicrobial agents to treat serious infections caused by multi-drug resistant bacterial pathogens. However, the co-occurrence of tigecycline and carbapenem resistance determinants challenges the clinical efficacy of these antimicrobial agents. In this study, we report the co-existence of *tet*(X4), *bla*_NDM−1_ and *bla*_OXA−58_ genes in the porcine *Acinetobacter towneri* isolate 19110F47. Sequence analysis revealed that *tet*(X4) gene, along with the florfenicol resistance gene *floR*, was flanked by three copies of IS*91*-like elements, which can form three different translocatable units (TUs), and were located in a 41,098-bp multidrug resistance region (MDRR) within a novel 100,354-bp genomic island (GI) region. TUs comprising *floR*-*vir*D2-IS*Vsa3, hp-abh-tet*(X4)-IS*Vsa3* and *virD2*-*floR*-IS*Vsa3*-*hp-abh*-*tet*(X4)-IS*Vsa3* can be looped out from the chromosomal DNA and facilitate the transfer of the TU-based resistance genes into other plasmidic or chromosomal sites. In addition, the carbapenemase genes *bla*_NDM−1_ and *bla*_OXA−58_ were found on different non-conjugative multiresistance plasmids in this isolate, with the genetic contexts IS*Aba125*-*bla*_NDM−1_-*ble*_MBL_-*tnpR* and ΔIS*Aba3-bla*_OXA−58_*-*IS*Aba3*, respectively. The simultaneous occurrence of *tet*(X4), *bla*_NDM−1_ and *bla*_OXA−58_ in the same porcine *A. towneri* isolate emphasizes the importance of antimicrobial resistance surveillance in food-producing animals.

## Introduction

Antimicrobial resistance poses a significant threat to public health globally. The presence of extensively drug-resistant (XDR) Gram-negative bacteria, in particular carbapenem-resistant *Enterobacteriaceae* and *Acinetobacter* spp., compromises the efficacy of carbapenems. Moreover, the choices of effective antimicrobial agents against carbapenem-resistant bacteria are very limited (1). Tigecycline has been recognized as a last-resort antimicrobial agent to treat infections caused by XDR Gram-negative bacteria (2). However, a variety of plasmid-borne *tet*(X) variants genes, which confer high-level resistance to tigecycline, have been reported in *Acinetobacter* spp. and *Enterobacteriaceae* from China (3, 4). The gene *tet*(X4) was detected in bacteria from food-producing animals, meat for human consumption, migratory birds, humans and environmental samples (5–7).

*Acinetobacter* spp. are considered as ubiquitous in the nature and have emerged as a major cause of nosocomial infections globally in recent decades (8). In addition, *Acinetobacter* spp. are not only associated with hospital-acquired infections, but also responsible for community-acquired infections (9). XDR *Acinetobacter* isolates, especially when they are carbapenem-resistant, are recognized as one of the most troublesome pathogens worldwide (10). Food-producing animals have been regarded as a potential reservoir for *Acinetobacter* spp. in many countries (11). To date, the *tet*(X4) gene was mostly reported in *E. coli*, and sometimes described in other bacterial species, such as *Acinetobacter* spp. (4, 12, 13). Currently, the genetic basis for co-resistance against tigecycline and carbapenems has been investigated in *Acinetobacter* spp. The tigecycline resistance genes *tet*(X) have been reported with carbapenem resistance gene *bla*_NDM_ in *A. baumannii, A. indicus, A. schindleri, A. lwoffii*, and other *Acinetobacter* (14–16).

In this study, we investigated a tigecycline- and carbapenem-resistant *A. towneri* isolate collected from a pig in China for the tigecycline- and carbapenem resistance genes present, their association with mobile genetic elements and their transfer potential.

## Materials and methods

### Sample collection and bacterial isolation

A total of 1,146 non-duplicate anal swab samples were collected from three unrelated and geographically distant pig farms and one pig slaughterhouse located in the Henan Province of China in 2019. Brain heart infusion (BHI) broth was used as transport medium for the anal swabs. The swabs were streaked on BHI agar plates supplemented with tigecycline (2 mg/L) and meropenem (2 mg/L) and incubated at 37°C for 24 h. Bacteria growing on these double-selective plates were identified to the species level by 16S rRNA amplification and sequencing of the amplicons (17).

### Antimicrobial susceptibility testing

The minimal inhibitory concentrations (MICs) of the *A. towneri* isolate to meropenem, tigecycline, ceftazidime, florfenicol, tetracycline, colistin and gentamicin were determined and evaluated using broth microdilution according to the recommendations of the Clinical and Laboratory Standards Institute (CLSI) (18). *E. coli* ATCC25922 was used as quality control strain.

### Whole-genome sequencing analysis

The *A. towneri* isolate 19110F47, was sequenced by using the PacBio RS and Illumina MiSeq platforms (Shanghai Personal Biotechnology Co., Ltd, China). The PacBio long reads were assembled with HGAP4 and CANU (Version 1.6) and corrected by the Illumina MiSeq short reads with pilon (Version 1.22). The prediction of ORFs and their annotations were performed using Glimmer 3.0. The blast software is used following the procedures at https://blast.ncbi.nlm.nih.gov.

### PCR analysis

The presence of translocatable units (TUs) was detected by PCR using the primers shown in Table 1. All the PCR products were subjected to Sanger sequencing. The obtained sequences were analyzed by BLAST comparison with the NCBI database (https://blast.ncbi.nlm.nih.gov/Blast.cgi).

**Table 1:** Primers used in this study.

**Primers**	**5^′^-3^′^**
TU1-rv	ACGACGCCCGCTATGATCCAA
TU1-fw	AACGCGGCACGTATAGGAAG
TU2-rv	AGTCCAACGGGTCCACCAC
TU2-fw	TGCTCATTTGATGCCTCCTT
TU3-rv	ACTTAAGGGCTATCTTGTTG
TU3-fw	TCATGGGATTTCTCGACCAC

### Transfer experiments

Conjugation assays were performed to assess the transferability of carbapenem and tigecycline resistance genes from *A. towneri* isolate to the azide-resistant recipient *E. coli* J53 and the rifampicin-resistant recipient *E. coli* EC600 according to a previously described method with a minor modification (1). Briefly, the donor and recipient strains were mixed at a ratio of 1:4 and incubated on LB agar for 5 h. The mixtures were collected and then plated on LB agar containing two selective markers, including azide (128 μg/mL) and meropenem (2 μg/mL), rifampicin (64 μg/mL) and meropenem (2 μg/mL), azide (128 μg/mL) and tigecycline (2 μg/mL), rifampicin (64 μg/mL) and tigecycline (2 μg/mL), respectively. The transconjugants were confirmed by PCR analysis.

## Results

### Identification of a carbapenem- and tigecycline-resistant *A. towneri* isolate

An isolate conferring resistance to both meropenem and tigecycline, designated 19110F47, was identified from swine origin in 2019. 16S rRNA sequence analysis suggested that it was assigned to the species *A. towneri*, which is involved in nosocomial infections as described in a previous study (16). Antimicrobial susceptibility testing results revealed that it had an expanded resistance profile, including resistances to meropenem (MIC, 16 μg/mL), tigecycline (8 μg/mL), ceftazidime (256 μg/mL), tetracycline (32 μg/mL), gentamicin (64 μg/mL) and florfenicol (128 μg/mL). However, it was classified by the CLSI breakpoints as colistin-intermediate (< 0.5 μg/mL). It is noteworthy that CLSI classifies all isolates with colistin MICs of ≤ 2 μg/mL as intermediate and does not provide a breakpoint for the category susceptible. Instead, when applying the breakpoints of the European Committee on Antimicrobial Susceptibility Testing (EUCAST) (http://www.eucast.org), this isolate would have been classified as colistin-susceptible.

### *tet*(X4) was located in the chromosomal DNA in *A. towneri* and is part of TUs

**Figure 1:**
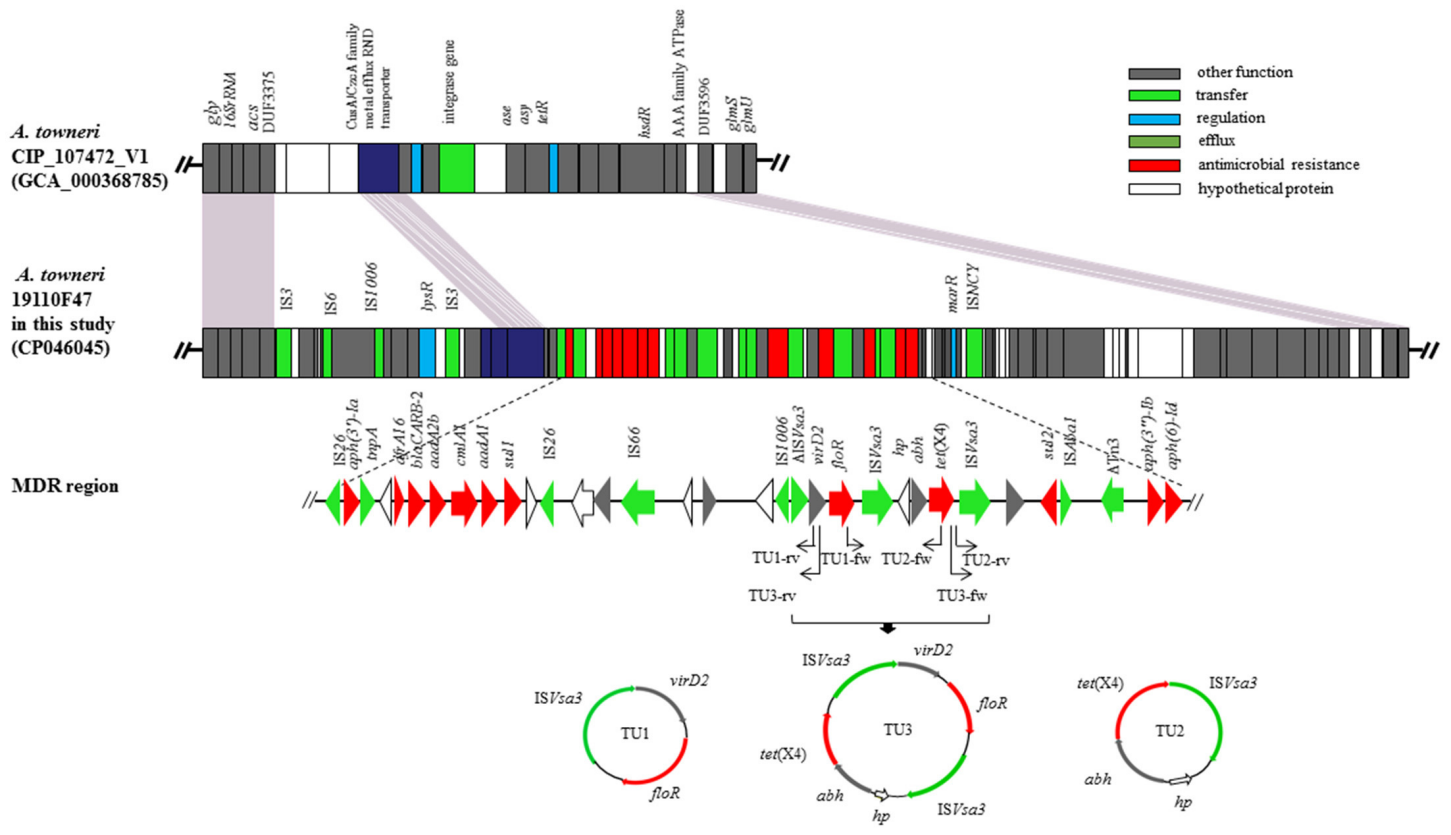
The genomic island carrying of the *tet*(X4) gene in the chromosomal DNA of *A. towneri* 19110F47 in this study. Genes are indicated by different colors. Δ indicates that the gene is truncated. The primers used for PCR were shown by arrows. Three TUs, including TU1 (*vir*D2-*floR*-IS*Vsa3*, 4,274 bp), TU2 [*hp-abh-tet*(X4)-IS*Vsa3*, 4,608 bp] and TU3 [*vir*D2-*floR*-IS*Vsa3*-*hp*-*abh-tet*(X4)-IS*Vsa3*, 8,882 bp] were formed.

The *tet*(X4) gene was found to be a part of a multidrug resistance region (MDRR) located on a 100,354-bp genomic island (GI) that was present in the chromosomal DNA of *A. towneri* 19110F47. The complete GI shared only a low query coverage (16–37%) with the sequences deposited in GenBank. It was inserted between the DUF3375 and AAA family ATPase encoding genes in the chromosomal DNA of *A. towneri* 19110F47. Database searches identified a distinctly smaller GI without an MDR region being present at the same location in *A. towneri* CIP_107472_V1 (accession number GCA_000368785.1) from an activated sludge plant in Australia. A total of 94 ORFs were identified in the GI of the present study. These ORFs encoded proteins responsible for several functions, such as gene regulation, transfer, efflux and antimicrobial resistance (Figure 1). The 41,098-bp MDRR consists of 33 ORFs [from IS*26* to *aph(6)-*Ib] (Figure 1). Among them, a total of 12 resistance genes were identified, including *tet*(X4), the aminoglycoside resistance genes *aadA1, aadA2b, aph(3”)-Ib, aph(3')-Ia* and *aph(6)-Id*, the sulfonamide resistance genes *sul1* and *sul2*, the phenicol resistance genes *cmlA1* and *floR*, the β-lactam resistance gene *bla*_CARB−2_ and the trimethoprim resistance gene *dfrA16* (Figure 1). In the close vicinity of *floR* and *tet*(X4), one truncated and two complete copies of IS*Vsa3*, all in the same orientation, were found (Figure 1), which was similar to the corresponding region on p47EC from a porcine *E. coli* (MK134376).

Three PCR assays were developed to detect IS*Vsa3*-mediated rolling-circle transposition of *tet*(X4) and/or *floR*. The results revealed that three TUs, including TU1 (*virD2*-*floR*-IS*Vsa3*, 4,274 bp), TU2 [*hp-abh-tet*(X4)-IS*Vsa3*, 4,608 bp] and TU3 [*virD2*-*floR*-IS*CR2*-*hp-abh-tet*(X4)-IS*Vsa3*, 8,882 bp] were formed in *A. towneri* 19110F47 (Figure 1).

### *bla*_*NDM*−1_ and *bla*_*OXA*−58_ were located on novel non-conjugative plasmids

**Figure 2:**
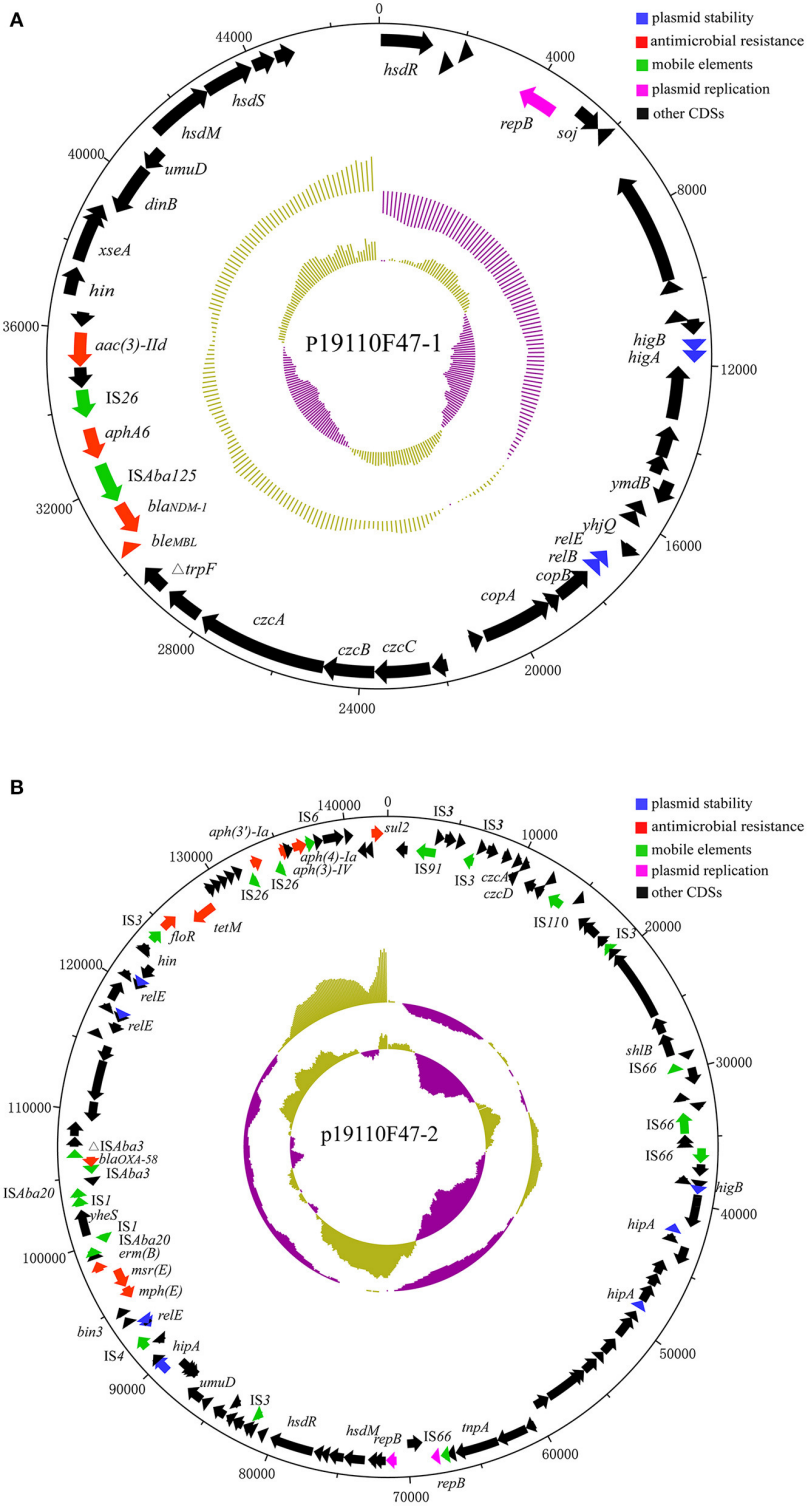
Structure of plasmids p19110F47-1 **(A)** and p19110F47-2 **(B)** from *A. towneri* 19110F47. The size scales are given in bp; genes are color-coded, depending on functional annotations: red, antimicrobial resistance; blue, plasmid stability; green, mobile elements; purple, plasmid replication; and black, other coding sequences (CDS).

The *bla*_NDM−1_ gene was located on a plasmid with a size of 47,094 bp, designated p19110F47-1 (Figure 2A). BLAST analysis revealed that p19110F47-1 showed similiarities with two other plasmids, pAT232 (GN014838) and pGX7 (CP071772), present in the GenBank database. However, these similarities did neither include the regions covering the antimicrobial resistance genes, nor those with the plasmid replication gene. Overall, a low query coverage (with the highest of 48%) (Supplementary Figure 1) was detected, suggesting that p19110F47-1 is a novel carbapenem resistance plasmid. Further analysis of the flanking regions of *bla*_NDM−1_ revealed that the insertion sequence IS*Aba125* was located upstream of *bla*_NDM−1_. Moreover, the *bla*_NDM−1_ gene was part of a region that contained also the resistance genes *aac(3)-IId, aphA6* and *ble*_MBL_ (Figure 2A).

The *bla*_OXA−58_-carrying plasmid p19110F47-2 was 143,035 bp in size, also carried the aminoglycoside resistance genes *aac(3)-IV, aph(3')-Ia* and *aph(4)-Ia, floR, sul2*, the tetracycline resistance gene *tet*(M), the macrolide-lincosamide-streptogramin B resistance gene *erm*(B), and the macrolide resistance genes *mph*(E) and *msr*(E) (Figure 2B). GenBank database searches identified the complete sequences of seven plasmids from *Acinetobacter* spp. with p19110F47-2 query coverage ranges from 40 to 87% (Supplementary Figure 2), and most of these plasmids harbored both *bla*_OXA−58_ and *tet*(X) orthologs (19, 20).

Conjugation experiments were performed using *A. towneri* 19110F47 as donor and two different *E. coli* strains as recipients. No transconjugants were obtained, which might be explained by the fact that the *bla*_NDM−1_-, but also the *bla*_OXA−58_-carrying plasmids lack a conjugative transfer region (Figure 2).

## Discussion

*Acinetobacter* spp. are ubiquitous in the natural environment, and have become important opportunistic pathogens, e.g., *A. towneri, A. baumannii, A. indicus, and A. lwoffii. Acinetobacter* spp. strains are widely distributed in a variety of environmental sources, including water, soil, foods, and livestock (15, 16, 21). In this study, we report, the identification of an *Acinetobacter* spp. strain co-harboring *bla*_NDM−1_, *bla*_OXA−58_ and *tet*(X4) collected from food-producing animals in China. It belonged to *Acinetobacter towneri*, which is involved in nosocomial infections (16). Carbapenem-resistant *Acinetobacter* spp. is one of the most dangerous pathogens in the world (21). And *Acinetobacter* spp. was the major reservoir of tigecycline-resistant *tet*(X) genes (12). The co-location of *tet*(X) and carbapenem resistance gene *bla*_NDM−1_ was previously described in *Acinetobacter* isolates from animals and the environment (14, 22). The strain 19110F47 had an expanded resistance profile. More attention should be paid to multidrug resistant (MDR) *A. towneri* isolates, because they have been reported increasingly in recent years, especially in hospital sewage and from livestock (23–26).

The *tet*(X4) gene conferring resistance to tigecycline was found to be on the chromosome of 19110F47. The *floR* and *tet*(X4) were found to be flanked by one truncated and two complete copies of IS*Vsa3*. IS*Vsa3* is 977 bp in size and represents a member of the IS*91* family. This family of insertion sequences differs from others in that they transpose by rolling circle transposition (27). Members of the IS*91* family can mobilize genes, including resistance genes that are located in their close vicinity. For this, only a single copy of the IS*91* element—not two copies as with most other insertion sequences—is necessary (27). The *tet*(X4) is the part of active TUs in the chromosomal DNA, may excise from the chromosomal DNA and transfer to other plasmidic or chromosomal sites. Of three TUs identified in *A. towneri* 19110F47, a sequence indistinguishable from that of TU2 was also found in p47EC (MK134376) from *E. coli* in a previous study (3), suggesting its mobility across genus boundaries. Of note, TU3 contained both *tet*(X4) and *floR*, implying that tigecycline resistance can be co-selected by florfenicol, a veterinary antimicrobial agent commonly used in cattle, pigs, poultry and fish (28).

The *bla*_NDM−1_ located on the plasmid p19110F47-1. Insertion of *bla*_NDM−1_ together with IS*Aba125* have been found within the chromosomal DNA and plasmids of *Acinetobacter* isolates (29, 30), suggesting that IS*Aba125* might be involved in the dissemination of *bla*_NDM−1_ among *Acinetobacter* spp. Numerous studies have documented that the *bla*_NDM−1_ was frequently present adjacent to truncated or intact IS*Aba125* as well as in other *Enterobacteriaceae*, including *E. coli, Klebsiella pneumoniae, Proteus mirabilis* and *Salmonella enterica* (31–33), indicating that IS*Aba125* plays a vital role in the spread of *bla*_NDM−1_ among different species of bacteria.

The *bla*_OXA−58_ was located on another plasmid p19110F47-2. An analysis for the genetic environments of *bla*_OXA−58_ showed that a truncated IS*Aba3* element was located upstream of *bla*_OXA−58_ and a complete IS*Aba3* element downstream of it. The genetic context found in the present study was consistent with that in plasmid pLHC22-2-tetX-162k (CP084298) in a previous study (19). In addition, the presence of two intact IS*Aba3* copies in opposite orientation up-stream and down-stream of *bla*_OXA−58_ was described in a series of studies and led to the assumption that IS*Aba3* might play a role in the transfer of *bla*_OXA−58_ (22, 34). It has been documented that transformation of a cloned *bla*_OXA−58_ gene on a low-copy-number vector into a susceptible *A. baumannii* strain increased the MICs of carbapenems, but not to levels considered as resistant (35, 36). For clinical carbapenem resistance in *Acinetobacter* spp., other carbapenem resistance mechanisms in addition to OXA-58-like β-lactamases are required (35). In the present study, the *A. towneri* isolate 19110F47 showed resistance to meropenem with a MIC of 16 μg/mL. It was speculated that *bla*_NDM−1_ played a dominant role in conferring the meropenem resistance phenotype.

The *tet*(X4), *bla*_NDM−1_ and *bla*_OXA−58_ genes have been documented in multiple studies solely or any two of them (14, 16, 20, 22, 37). Only one study by Zheng *et al*. described that a *tet*(X6) variant and the two carbapenemase genes *bla*_NDM−1_ and *bla*_OXA−58_ were located on the same plasmid in an *A. baumannii* isolate of chicken origin (15). In the current study, we identified the occurrence of *tet*(X4), *bla*_NDM−1_ and *bla*_OXA−58_ in the same *A. baumannii* isolate of swine origin, with *tet*(X4) being located on a novel chromosomal GI and *bla*_NDM−1_ and *bla*_OXA−58_ carried by two novel plasmids.

## Conclusions

In conclusion, we described the co-existence of *tet*(X4), *bla*_NDM−1_ and *bla*_OXA58_ in a porcine *A. towneri* isolate that displays resistance to carbapenems and tigecycline among numeous other antimicrobial agents. The *tet*(X4) is part of active translocatable units in the chromosomal DNA, facilitating its transfer into other plasmidic or chromosomal sites. The emergence of chromosomal *tet*(X) genes combined with plasmid-mediated carbapenem resistance genes in the same isolate, as shown in this study, will further compromise the treatment options of severe infections caused by *Acinetobacter* spp.

## Data availability statement

The datasets presented in this study can be found in online repositories. The names of the repository/repositories and accession number(s) can be found in the article/supplementary material.

## Author contributions

X-DD and HY designed the research and supervised the study. AL, WZ, RY, and CL performed the experiments and analyzed the data. AL and HY wrote the manuscript. SS and X-DD reviewed and edited the manuscript. All authors revised the manuscript and approved the final version for submission.

## Funding

This work was supported by Henan Outstanding Foreign Scientist Studio (No. GZS2022010), High-end Foreign Experts Project (G2022026023L), the China Postdoctoral Science Foundation (No. 2021M690923), and the Federal Ministry of Education and Research (BMBF) under project number 01KI2009D as part of the Research Network Zoonotic Infectious Diseases.

## Conflict of interest

The authors declare that the research was conducted in the absence of any commercial or financial relationships that could be construed as a potential conflict of interest.

## Publisher's note

All claims expressed in this article are solely those of the authors and do not necessarily represent those of their affiliated organizations, or those of the publisher, the editors and the reviewers. Any product that may be evaluated in this article, or claim that may be made by its manufacturer, is not guaranteed or endorsed by the publisher.

## Supplementary material

Supplementary Figure 1 Structure analysis of *bla*_NDM−1_-bearing plasmid. Comparison analysis of the plasmid p19110F47-1 with other similar plasmids submitted in GenBank database.

Supplementary Figure 2 Structure analysis of *bla*_OXA−58_-bearing plasmid. Comparison analysis of the plasmid p19110F47-2 with other similar plasmids submitted in GenBank database.
