# Supplementary figures and images for: Characterization of a genomic Island carrying the tet(X4) gene in porcine Acinetobacter towneri co-harboring plasmid-borne blaNDM−1 and blaOXA−58 genes
Source: Front Vet Sci. 2022 Sep 29;9:1002149. doi: 10.3389/fvets.2022.1002149 (PMC9557058; doi:10.3389/fvets.2022.1002149)

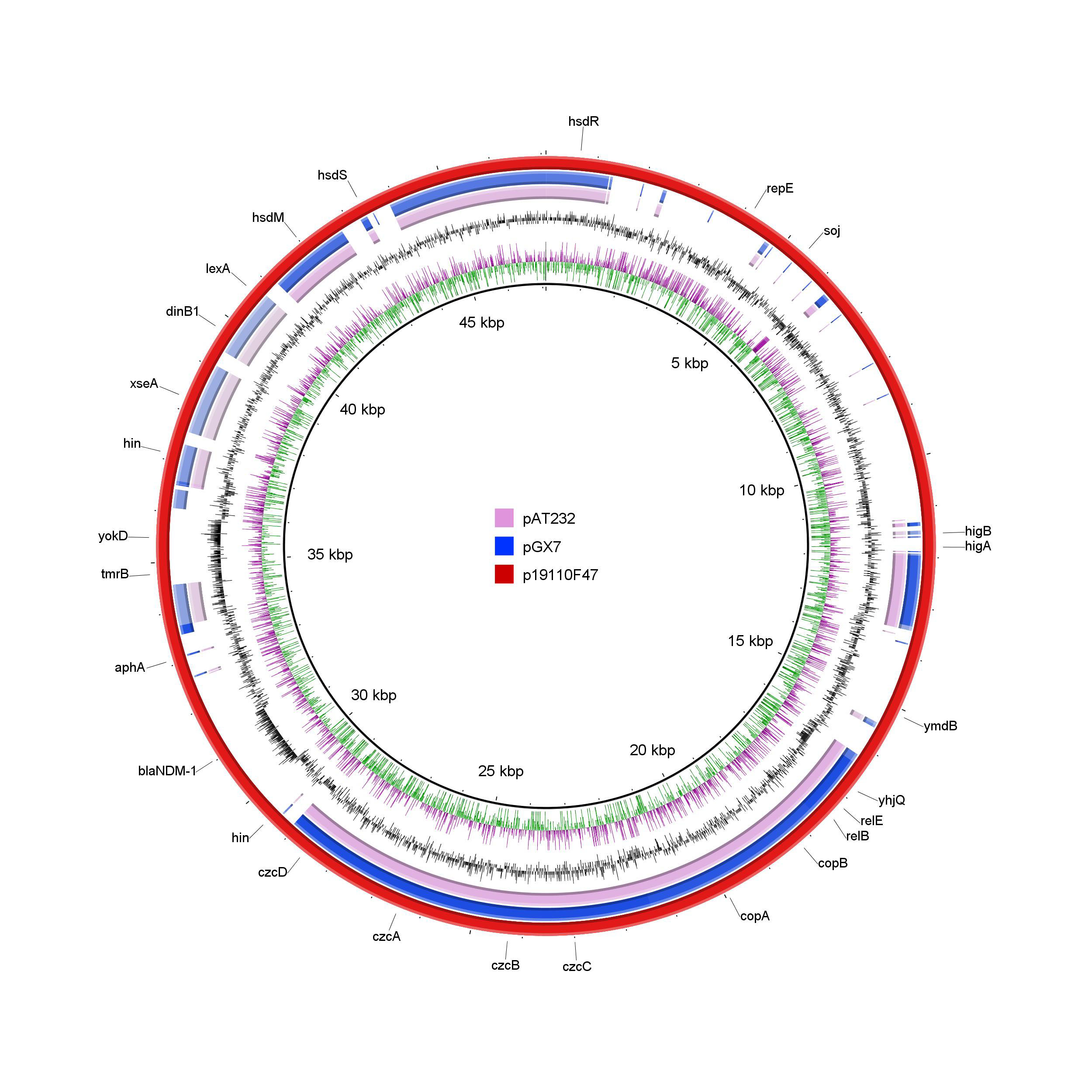

Supplement: Supplementary Figure 1 — Structure analysis of blaNDM−1-bearing plasmid. Comparison analysis of the plasmid p19110F47-1 with other similar plasmids submitted in GenBank database. [file Image_1.JPEG]

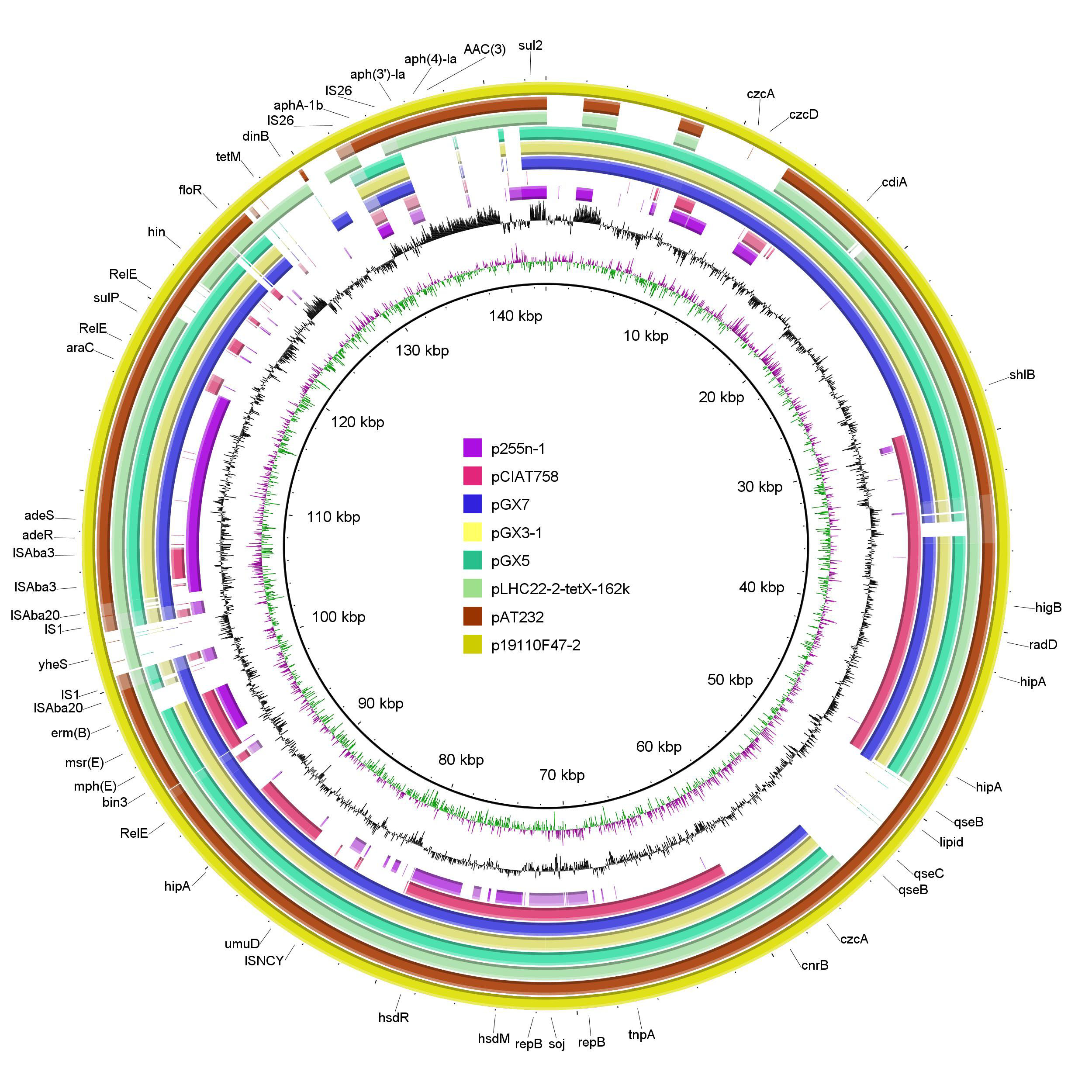

Supplement: Supplementary Figure 2 — Structure analysis of blaOXA−58-bearing plasmid. Comparison analysis of the plasmid p19110F47-2 with other similar plasmids submitted in GenBank database. [file Image_2.JPEG]
